# ECR-MAPK Regulation in Liver Early Development

**DOI:** 10.1155/2014/850802

**Published:** 2014-12-18

**Authors:** Xiu-Ju Zhao, Hexian Zhuo

**Affiliations:** ^1^School of Biology and Pharmaceutical Engineering, Wuhan Polytechnic University, Collaborative Innovation Center of Processing of Agricultural Products in Hubei Province, No. 68 South Xuefu Road, Changqing Garden, Wuhan 430023, China; ^2^Wuhan Institute of Physics and Mathematics, Chinese Academy of Sciences, West No. 30 Xiaohongshan, Wuhan 430071, China; ^3^Xinxiang Institute for Drug Control, No. 17 Jiankang Road, Xinxiang 453000, China

## Abstract

Early growth is connected to a key link between embryonic development and aging. In this paper, liver gene expression profiles were assayed at postnatal day 22 and week 16 of age. Meanwhile another independent animal experiment and cell culture were carried out for validation. Significance analysis of microarrays, qPCR verification, drug induction/inhibition assays, and metabonomics indicated that *alpha-2u globulin* (extracellular region)*-socs2* (-SH_2_-containing signals/receptor tyrosine kinases)*-ppp2r2a/pik3c3* (MAPK signaling)*-hsd3b5/cav2* (metabolism/organization) plays a vital role in early development. Taken together, early development of male rats is ECR and MAPK-mediated coordination of cancer-like growth and negative regulations. Our data represent the first comprehensive description of early individual development, which could be a valuable basis for understanding the functioning of the gene interaction network of infant development.

## 1. Introduction

Development is differential expression of the genome of organisms in different time points. Mammalian liver especially plays a vital role in the coordination of various physiological processes, and due to the different metabolic needs for male and female reproduction, mammalian liver shows considerable sexual dimorphism; this phenotypic expression is mediated via sex hormones [1]. Androgen response of the rat liver shows notable change during individual development and only the postpubertal adult (40–750 days of age) is subject to androgen-inducible genes and androgen-repressible genes [2]. Thus, transcriptional control in mammals must be properly coregulated for early stages of liver formation, perinatal repression, and position-dependent regulation [3]. Furthermore, expression profiles of fetal and natal liver tissues from mice reveal two stages during embryonic liver development; embryonic day 14.5 is a transition point when hepatocytes occur. Postnatal processes are also divided into two stages (*Ι* *Ι* *Ι* and *Ι*V) and genes expression profile of stage *Ι*V (ranging from day 7 to week 18) exhibited more invariant property [4].

Previous researches focus on embryonic development, using targeted methods such as genetic modification, quantitative PCR, hybridization, and electrophoresis. However, early growth is less concerned. Early growth consists of natal, prepuberty, puberty, and adult stages. And moreover, early growth is connected to a key link between embryonic development and aging [4]. Therefore it is necessary to deepen the study of the early development of individual growth and thus to provide a basis and reference for dietary intake and disease prevention and control in the process of human growth, especially infant.

Utilizing systems biology approaches, for example, by combining global gene expression profiling and metabolic pattern techniques, provides means to determine characteristic transcript profile and endpoint metabolic effects of development. Integrated information from transcriptomic and metabonomic profiling contributes to elucidate mechanisms of a developmental effect in detail and with comprehensiveness.

This research compared the gene expression profiles of 22 days (3 weeks) and 16 weeks of age, using Wistar rat as a model from public database, and furthermore clinical biochemistry, qPCR, cell culture, and NMR were carried out for validation and confirmation from independent animal experiment, to reveal temporal migration information and the transcription pattern of this early growth process.

## 2. Materials and Methods

### 2.1. Animal Experiment and Cell Culture

This study partially came from public database GSE32156 [5]. Briefly, offspring from Wistar Han dams were fed normally. Liver samples were collected at postnatal day (PND) 22 (*n* = 5) and week 16 (*n* = 5) of age for liver gene expression profile analysis. Independent animal experiment was carried out according to guidelines of the government of China. Sera for clinical biochemistry, urines for NMR, and livers for qPCR and cell culture were collected when the rats were decapitated after anesthesia with isoflurane. Rat primary liver cells were cultured and dexamethasone (dex, 0.1 *μ*M) or cycloheximide (CHX, 0.05 *μ*g/mL) was added as indicated.

### 2.2. Clinical Biochemistry

Sera were analyzed for glucose (Glc), total cholesterol (CHOL), creatinine (CREA), triglyceride (TG), albumin (ALB), aspartate aminotransferase (AST), alanine aminotransferase (ALT), alkaline phosphatase (AlkP), total protein (TP), and testosterone using biochemical analyzer and radioimmunoassay.

### 2.3. Transcriptomic Analysis

Total RNA was isolated from 10 rat livers, 5 from the control pups and 5 from the adults, with Trizol Reagent (Invitrogen Corp., Carlsbad, CA), in accordance with the manufacturer's instructions. The concentration and purity of total RNA were determined by spectrophotometer, 28S and 18S rRNA. The Affymetrix Rat Genome 230 2.0 arrays were used to monitor variations in gene expression profile. The log-transformed (base = 2) data were obtained for all probes and array-wise normalized using Affymetrix Dchip 2006.

The* t*-test and Wilcoxon signed-rank test were utilized for significance analysis of microarrays (SAM) [6–9]. A permutation test was employed for estimating the false-discovery rate (FDR < 0.05, *n* = 200~500). The CapitalBio Molecule Annotation System (MAS), KEGG, and GenMAPP databases were used for pathway analysis (http://bioinfo.capitalbio.com/mas). For each pathway, genes with known rat orthologues were compared with sets of significant genes from SAM to define the effects of corresponding pathway.

The relationship of genes or gene clusters was carried out using Pearson's correlation, Spearman's correlation, or 2D STOCSY (statistical total correlation spectroscopy).

### 2.4. Quantitative Real-Time PCR and Western Blot

cDNA was synthesized using an oligo-(dT)15 primer (Invitrogen). PCR primers were designed with Primer Premier 5.0 software. The housekeeping gene*β*-actin was used as an internal control. The PCR amplification was conducted at 95°C for 15 min, followed by 40 cycles of 94°C for 5 s, 58°C for 15 s, and 72°C for 10 s. The relative mRNA levels of selected genes were calculated with the 2^−ΔΔCt^ method [10]. Liver proteins were subjected to sodium dodecyl sulfate-polyacrylamide gel electrophoresis and transferred to blotting membrane. Immunoblots were blocked with 3% bovine serum albumin in Tris-buffered saline/Tween-20 buffer for 60 min at room temperature and incubated overnight at 4°C with primary antibodies. Blots were developed by an Enhanced Chemiluminescence Western blotting kit (Amersham Biosciences, Uppsala, Sweden) and visualized by a Gene Genome bioimaging system. Bands were analyzed by densitometry with GeneTools software (Syngene, Frederick, MD, USA). Values were reported as means ± SD. Statistical differences were determined by the one-way ANOVA multiple range test and the Wilcoxon rank sum test. Statistical significance was set at *P* < 0.05.

### 2.5. NMR Spectroscopy Acquirement

550 *μ*L urine was mixed with 55 *μ*L of phosphate buffer, followed by centrifugation. 1D ^1^H NMR spectra were acquired (298 K, Bruker Avance III-600 MHz NMR spectrometer) with 32 transients for urine using a standard presaturation pulse sequence (presaturation during a relaxation delay and during the mixing time). 2D J-resolved, ^1^H-^1^H correlation, total ^1^H-^1^H correlation, ^1^H-^13^C heteronuclear single quantum coherence, and ^1^H-^13^C heteronuclear multiple bonds correlation NMR spectra [11–13] were acquired for selected urine to assign metabolites.

### 2.6. Statistical Analysis of NMR Spectra

NMR spectra were processed routinely [14] for phase, baseline, and chemical shift reference calibrations.

Unsupervised PCA (principal component analysis) was performed (SIMCA-P 11.0 demo, Umetrics, Sweden) to outline intrinsic similarity/dissimilarity within the data set scaled to unit variance (UV). Comparisons between temporal animals were made by carrying out qualitative PLS (partial least square regression) and O-PLS (orthogonal projection to latent structures) models (class information as qualitative* Y* variable). The validity of the models was assessed by *Q* ^2^ (predictability) and *R* ^2^ (interpretability) of the model. Meanwhile, the same models were validated by a 7-fold cross validation [15], cross validation-ANOVA [16–18], and a permutation test (*n* = 200) [19]. Valid models including significantly changed metabolites (denoted by red color) were visualized and shown in the colored correlation coefficient loading plots (MATLAB version 7.1, Mathworks Inc; Natwick, USA).

## 3. Results

### 3.1. Weight and Clinical Biochemistry of Early Individual Development

Adult rats (~313.6 g) have much more weight than pups (~219.3 g) (*P* < 0.001, Table 1). Serum clinical biochemistry data from the adult rats contained higher levels of metabolites, such as glucose, triglyceride, testosterone, and lower enzymes, such as aspartate aminotransferase (AST), alanine aminotransferase (ALT), and alkaline phosphatase (AlkP) compared with those from the pup rats (*P* < 0.05, Table 1).

### 3.2. Coexpressing Genes of Early Individual Development

Coexpressing genes between adults and pups belong to REDOXIDATION [ATP binding, electron carrier activity, oxoglutarate dehydrogenase (succinyl-transferring) activity, L-2-hydroxyglutarate dehydrogenase], PROTEIN TRANSLATION, TRANSLOCATION AND PROTEOLYSIS [ADP-ribosylation factor binding, glutamyl-tRNA aminoacylation, proteolysis, protein tyrosine phosphatase activity, SMAD protein nuclear translocation], TRANSCRIPTION REGULATION [ADP-ribose diphosphatase nucleotide and nucleic acid metabolic process, 3′ pre-RNA cleavage], CELL CYCLE [nucleotide-excision repair (cyclin-dependent protein serine/threonine kinase activity), positive regulation of cell proliferation], and SYSTEM ORGANIZATION [acrosome intracellular phorbol ester receptor signaling cascade, nervous system development, motor axon guidance, neurotransmitter acetylcholine receptor activity, G protein coupled olfactory receptor protein signaling pathway, detection of chemical stimulus involved in sensory perception of bitter taste, keratin filament, regulation of the force of heart contraction, regulation of hindgut contraction] (Supplementary Table 1, *P* > 0.965, 0.9999 ≦ change fold ≦ 1.0001) (see Supplementary Table 1 in Supplementary Material available online at http://dx.doi.org/10.1155/2014/850802).

### 3.3. Differential Genes of Early Individual Development

The differential genes between adults and pups were listed (Table 2), of which 48 genes/probe sets were upregulated (*P* < 0.05, fold > 1.53) and 30 genes were downregulated (*P* < 0.05, fold < 0.665). According to GO terms [20], these BIOLOGICAL PROCESSES were transport, C21-steroid hormone biosynthetic process, metabolic process, oxidation reduction, estrogen metabolic process, digestion, cell morphogenesis, oxygen and reactive oxygen species metabolic process, superoxide metabolic process, transcription, cell proliferation, cell differentiation, protein amino acid dephosphorylation, regulation of cell growth, cell adhesion, immune response, and proteolysis; CELL COMPONENTS were extracellular region and space, mitochondrion, endoplasmic reticulum, peroxisome, integral to membrane, protein phosphatase type 2A complex, plasma membrane, and Golgi-associated vesicle; MOLECULAR FUNCTIONS were transporter activity, catalytic activity, 3-beta-hydroxy-delta-5-steroid dehydrogenase activity, monooxygenase activity, endopeptidase inhibitor activity, estrone sulfotransferase activity, estradiol 17-beta-dehydrogenase activity, identical protein binding, glucuronosyltransferase activity, superoxide-generating NADPH oxidase activity, nucleic acid binding, methyltransferase activity, protein binding, protein tyrosine/serine/threonine phosphatase activity, oxidoreductase activity, protein phosphatase type 2A regulator activity, growth hormone receptor binding, proton-dependent oligopeptide secondary active transmembrane transporter activity, metalloendopeptidase activity, sterol/transporter activity, asparagine synthase (glutamine-hydrolyzing) activity, cytokine activity, transferase activity (transferring acyl groups other than aminoacyl groups), aldo-keto reductase activity, and so forth.

### 3.4. Pathway Significance of Early Individual Development

The significant pathways were as follows: transport (*obp3*(*AB039825*),* obp3*(*J00738*),* AB039826*,* AB039823*,* ust5r*,* mup5*,* AB039828*,* clca5*,* abcg8*,* abcd2*,* aqp7*, and* abcc3*; *P* = 0.001) and cell adhesion (*cdh17*,* omd*,* jam3*,* pcdh17*,* ncam2*,* amigo1*; *P* = 0.005); steroid biosynthesis (*obp3*,* hsd3b5*,* akr1c1*,* hsdl2*,* hsd17b6*,* hsd11b2*,* ar*, and* cyp17a1*;* P* = 8.6*e *− 9), metabolic process (*hsd3b5*,* dhrs7*,* hao2*,* mett17b*,* hsdl2*,* asns*,* acsm2*, and* psat1*; *P* = 0.035), oxidation reduction (*hsd3b5*,* cyp2c13*,* cyp3a9*,* cyp2a2*,* hao2*,* akr1c1*,* cyp2c29*,* nox4*,* rgd1564865*,* hsdl2*,* me3*,* cyp2c12*,* cyp17a1*, and* akr1b7*; *P* = 0.0001), and fatty acid biosynthesis (*scd*,* fasn*; *P* = 0.00014); transcription regulation (*zfp37*,* zfp68*,* npas2*,* taf9b*,* ppargc1a*, and* nfe2*; *P* = 0.0003) and regulation of cell cycle (*ccng2*,* ccnb2*, and* ccna2*; *P* = 0.00018); skeletal system development (*col4a1*,* col5a2*,* col5a1*,* col1a2*, and* col1a1*;* P* = 4.4*e* − 5) and organization (*cav2*,* pex11a*,* onecut1*,* meox2*,* cml3*,* col5a2*, and* lox*; *P* = 0.0051); immune response (*rt1-ce5*,* cxcl9*,* rt1-aw2*,* tnfsf13*, and* cxcl13*; *P* = 0.099), signaling (*ppp2r2a, socs2*,* olr59*,* rgs3*,* adora2b*,* cish*,* atp6ap2*,* pik3c3*,* ppp1r2*,* ghr*, and* nrg1*; *P* = 0.00067), and proteolysis (*mup5*,* mme*,* trhde*,* spink3*,* prcp*, and* rgd1562284*; *P* = 9.1*e* − 4) (Table 3).

### 3.5. Genes Correlation Network of Early Individual Development

**Figure 1:**
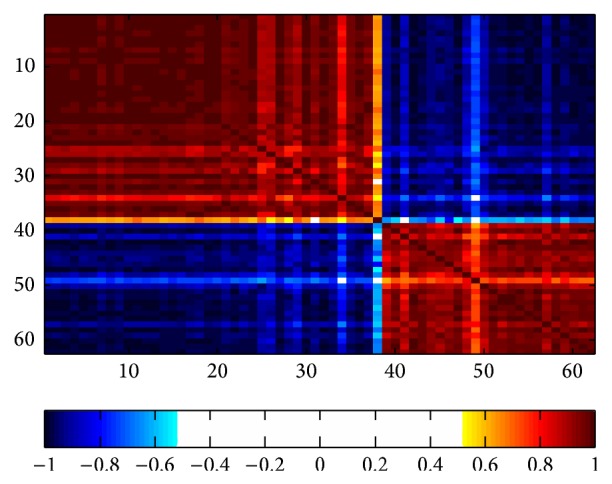
Gene correlation network of liver early development.* obp3*, extracellular region and transporter, was correlated to membrane (*ust5r, stac3, cdh17, mme, olr59, gpm6a, tmem163, abcg8, abcd2, *and* abcc3*), adapter (*stac3, socs2*), transcription (*zfp37, ccna2, asns, *and* rgd1562284*), immune (*rt1-ce5, rt1-aw2, cxcl13, *and* cyfip2*), and redox (*hsd3b5, cyp2c13, cyp2a2, dhrs7, hao2, akr1c1, nox4, inmt, dusp1, mettl7b, ppp2r2a, cyp17a1, *and* akr1b7*). Genes were correlated using 2D STOCSY, *P* < 0.05. Keys: 1,2 obp3; 3,4,7 rup2; 5, hsdsb5; 6, dhrs7; 8, cyp2c13; 16, ust5r; 19, stac3; 22, zfp37; 32, ppp2r2a; 33, socs2; 39, pcdh17; 41, abcg8; 44, ccna2; 49, s100g; 50, cxcl13; 53, tox; 62, akr1b7. The corresponding genes of numbers were listed in Supplementary Table  2.

Furthermore,* obp3*, extracellular region and transporter, was correlated to membrane (*ust5r*,* stac3*,* cdh17*,* mme*,* olr59*,* gpm6a*,* tmem163*,* abcg8*,* abcd2*, and* abcc3*), adapter (*stac3*,* socs2*), transcription (*zfp37*,* ccna2*,* asns*, and* rgd1562284*), immune (*rt1-ce5*,* rt1-aw2*,* cxcl13*, and* cyfip2*), and redox (*hsd3b5*,* cyp2c13*,* cyp2a2*,* dhrs7*,* hao2*,* akr1c1*,* nox4*,* inmt*,* dusp1*,* mettl7b*,* ppp2r2a*,* cyp17a1*, and* akr1b7*) (|*r* | >|*r*_cutoff_ | = 0.632, *P* < 0.05) (Figure 1, Supplementary Table 2).

### 3.6. qPCR Validation of Early Individual Development

**Figure 2:**
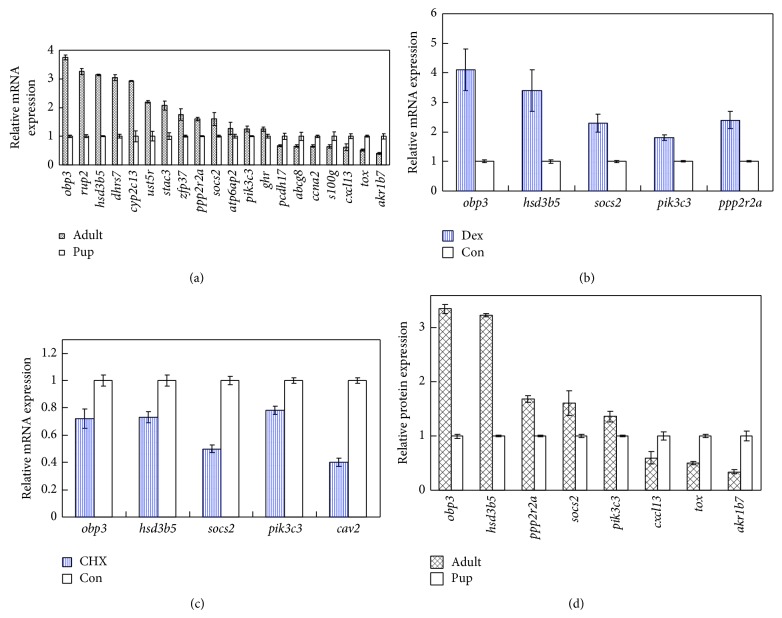
mRNA and protein relative expression of liver early development. (a) qPCR validation for highlighted microarray genes between pups and adults, (b) 0.1 *μ*M dexamethasone (dex) induction of obp3 and coupregulation of its targets of pups, (c) 0.05 *μ*g/mL cycloheximide (CHX) inhibition and codownregulation of related genes of pups, (d) Western blotting for key proteins between pups and adults. All genes/proteins are significant, *P* < 0.01.

qPCR validation for highlighted microarray genes was carried out for pups and adults. The results demonstrated that the mRNA level of* obp3*, a major regulator in odorant binding, was elevated 3.75-fold in the adult group compared to pups; the expression levels of* rup2, hsd3b5, dhrs7, cyp2c13, ust5r, stac3, zfp37, ppp2r2a, socs2, atp6ap2, pik3c3, *and* ghr* were elevated significantly in the adult rats compared to the pups, while the expression levels of* pcdh17, abcg8, ccna2, s100 *g*, cxcl13, tox, *and* akr1b7 *were decreased significantly (Figure 2(a), *P* < 0.01).

### 3.7. Induction/Inhibition of* obp3* and Its Targets

In order to characterize the interplay between* opb3* and its potential targets, we evaluated the dexamethasone induction/cycloheximide inhibition of* obp3* and coregulated genes of adults. Dexamethasone (0.1 *μ*M) induced* obp3* expression and upregulated its targets:* hsd3b5, socs2, pik3c3, ppp2r2a *(Figure 2(b)), and* obp3* expression inhibition (0.05 *μ*g/mL cycloheximide) downregulated* hsd3b5, socs2, pik3c3, *and* cav2 *(Figure 2(c)).

### 3.8. Obps and Its Related Proteins

In order to characterize opb3 protein and its related proteins, we assayed Western blotting. The expression levels of obp3, hsd3b5, ppp2r2a, socs2, and pik3c3 proteins were elevated significantly in the adult rats compared to the pups, while the expression levels of cxcl13, tox, and akr1b7 proteins were decreased significantly (Figure 2(d), *P* < 0.01).

### 3.9. Metabolic Profile of Temporal Rats

**Figure 3:**
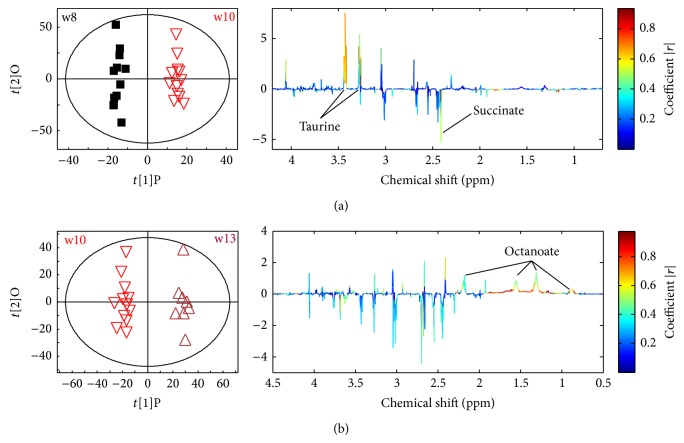
Taurine and octanoate increased with age. Cross validated qualitative O-PLS scores plots (left) and corresponding coefficient loading plots (right). Urine spectra comparison between w8 with w10 (a,* Q* ^2^ = 0.759) and w13 versus w10 (b,* Q* ^2^ = 0.992). Here, the red color indicated important discriminatory metabolites whereas the blue color indicated no significance in discrimination. w8: black boxes, w10: red inverted triangles, and w13: purple triangles.

Using PLS, invalid models indicated that adults were metabolically stationary from week 15 to week 19 of age (Table 4). With age (from week 8 to week 13 of age), taurine and octanoate (8 : 0) were increased, whilst succinate was lowered (*P* < 0.05, Figure 3).

## 4. Discussions

Early development is a physiology process, and we found that in this early individual development, extracellular region and space (ECR)—SH_2_ containing protein—MAPK pathway plays a vital role. Meanwhile, early individual development is ECR and androgen-mediated feedforward coordination network of positive cancer-like growth and negative regulations.

### 4.1. Androgen-Responsive Genes

Androgen-dependent *α*2u globulin (obp3) is a group of low molecular weight (Mr ~18,000) male specific urinary proteins synthesized and secreted by hepatocytes. In the male rat, hepatic synthesis of *α*2u globulin begins at puberty (~40 days), reaches a peak level (~20 mg/day) at about 75 days, and declines during old age [21]. Age-dependent changes in the expression of androgen-responsive genes (alpha 2u globulin) reflect changing androgen sensitivity [2].

Meanwhile, cell cycle and mitosis gene* mapre1* at week 16 was upregulated 1.25 times than at week 3 (*P* = 0.0026).

Accordingly,* androgen receptor* at week 16 was upregulated 1.25 times than at week 3 (*P* = 2.4*E* − 05).

### 4.2. Development Network

**Figure 4:**
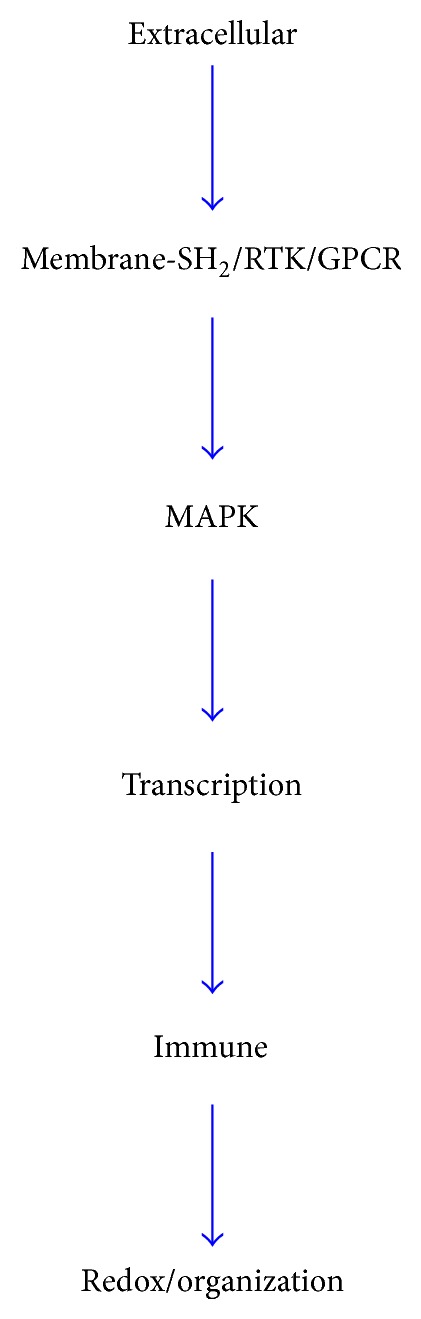
ECR-MAPK-mediated early individual development network. Extracellular region and space (ECR) act as nutrition ligand and information input. Ligands interact with membrane transports and SH_2_-containing/MAPK related signals and regulate cell cycle, transcription, and proteolysis, leading to short-term steroid, fatty acid biosynthesis, redox and metabolic process, and long-term collagen development and organization. G protein coupled receptors/G protein, catalytic receptors, and ECR signals converge at MAPK cascades.

Based on gene profiling, verification at mRNA, protein, and metabolite levels, we postulated that, in early development, extracellular region and space (ECR)* obp3, rup2, pcdh17, a2m, *and* cxcl13* act as nutrition ligand and information input. Ligands interact with membrane transports* ust5r, cdh17, mme, olr59, gpm6a, tmem163, abcg8, abcd2, abcc3,* and SH_2_-containing/MAPK related signals* stac3, socs2, cish*/*pik3c3, *and* nrg1* and regulate cell cycle, transcription, and proteolysis* ccng2, ccnb2, ccna2/zfp37, zfp68, npas2, taf9b, ppargc1a, nfe2/hspb1, usp18, mup5, mme, trhde, spink3, *and* prcp*, leading to short-term steroid, fatty acid biosynthesis, redox, and metabolic process* obp3, hsd3b5, akr1c1, hsdl2, hsd17b6, hsd11b2, ar, cyp17a1/scd, fasn/cyp2c13, cyp3a9, cyp2a2, hao2, cyp2c29, nox4, me3, cyp2c12, akr1b7/dhrs7, mett17b, asns, acsm2, psat1* and long-term collagen development and organization* col4a1, col5a2, col5a1, col1a2, col1a1/cav2, pex11a, onecut1, meox2, cml3, *and* lox*. G protein coupled receptors/G protein* olr59, rgs3 *[22]*, adora2b/gnai3, gnat3*, catalytic receptors* socs2 *[23]*, nim1 *[24]*, atp6ap2, ghr* and ECR signals converge at MAPK cascades (Figure 4). Protein expression to some extent confirmed key genes, for example, obp3, socs2, ppp2r2a, pik3c3, cxcl13, and hsd3b5 proteins dynamics (Figure 2).

### 4.3. Female-Prefer Genes

Female-specific* tox *changes in gene expression during postnatal liver development reflect the deceleration of liver growth and the induction of specialized liver functions, with widespread changes in sex-specific gene expression primarily occurring in male liver [25].

Male and female genes are both increased, but their magnitudes in male are larger than that in female-change fold of male gene* obp3* is 3.75 times more at senior than at junior.

### 4.4. Development and Cancer

Hsd3b5 expression showed significant associations with the degree of hepatic steatosis [26], accompanied by increased testosterone with age (Table 1). Expression level of dehydrogenase/reductase member 7 (*dhrs7*) in rat regenerating liver was more than 968-fold compared to control [27].* Cdh17 *[28]*, nim1 *[24]*, scd, *and* fasn *[29] were related to disease/cancer; upregulation of* fasn* was in accordance with elevated moderate-chain fatty acid octanoate (8 : 0). Thus, developmental process poses cancer-like characteristics.

Overlap between embryonic liver development and liver cancer is not only in cell cycle or apoptosis, but also in metabolic pathways associated with carbohydrate and lipid metabolism [30]. Fetal hepatocytes have high IGF2 and E2F3 expressions, and levels of IGF2 and E2F3 mRNA were positively correlated to human prostate and bladder cancers [31]. However, fetal and infant livers have no cancers.

### 4.5. Negative Control Genes

*Socs2* [23],* cdkn1a* [32],* rgs3* [22],* cish*,* spink3*,* cyp17a1*, and* nfe2* [33] were involved in negative control. Cancer-like early individual development, but no cancer, is maybe due to counteracting effects of negative control and cooperation of the two sides.

### 4.6. Feedforward Regulation

Feedforward regulation in pheromone-activated MAPK pathway ensures stability and rapid reversibility of a cellular state [34].

*Cxcl13*, belonging to extracellular region [35], takes part in positive regulation of cytosolic calcium ion concentration and immune response [36].

Nonzero uterus dependent initial conditions allow fast early development and sensing, and meanwhile, feedforward modulations appear at reversible developmental transitions, because this network control can obtain the aims of growth stability and rapid reversibility without loss of external signaling information [34].

In a summary, qPCR validation was for gene expression profile, and meanwhile, cell induction/inhibition assays, Western blot, and NMR-based metabonomics were carried out for confirmation of gene results. Using dynamic assays of body weight, serum biochemistry, transcript, protein, and metabolite profile, we reveal that, in early individual development, increasing magnitude in male is larger than that in female, and cancer-like growth coordinates negative regulation; meanwhile, feedforward modulations appear at developmental transitions, obtaining aims of growth stability and rapid reversibility without superoxidation or maglinant growth; more importantly, extracellular matrix-kinase cascade responses play a vital role in this early individual development. Taken together, extracellular matrix-kinase cascade-based feedforward cooperation of cancer-like growth and negative regulation realize win-win long-term growth stability and short-term rapid reversibility/fluctuation in gradual transition of early individual development. This finding is particularly important for understanding the gene expression network of infant development.

## Supplementary Material

Supplementary Table 1: Coexpressing genes between 16 weeks and 3 weeks old.
Supplementary Table 2: Correlation coefficients of genes network.

## Figures and Tables

**Table 1:** Body mass and biochemistry indices between adults and pups.

Index	Pup	Adult	*P*
Body mass, g	219.3 ± 8.8	313.6 ± 20.8	1.0*E* − 12
ALT, IU/L	37.5 ± 3.8	30.8 ± 3.9	0.00041
AST, IU/L	176.7 ± 22.2	160.2 ± 30.9	0.035
AlkP, IU/L	70.7 ± 12.9	41.6 ± 4.6	5.7*E* − 07
Glc, mmol/L	4.8 ± 0.5	5.5 ± 0.4	0.00035
TG, mmol/L	0.57 ± 0.13	0.76 ± 0.26	0.033
Testosterone, ng/mL	25 ± 3	40 ± 4	0.046

**Table 2:** Significantly changed genes between 16 weeks and 3 weeks old.

Public ID	Gene symbol	*P*	Fold
AB039825	*obp3 *	2.12*E* − 07	3.75
J00738	*obp3 *	6.23*E* − 07	3.45
AF368860	*loc680367 /// loc680406 /// rup2 *	2.97*E* − 06	3.40
AF198441	*rup2 *	0.000102	3.26
NM_012584	*hsd3b5 *	2.02*E* − 08	3.14
BI288203	*dhrs7 *	3.84*E* − 06	3.05
AF368860	*loc680367 /// rup2 *	0.000457	3.02
J02861	*cyp2c13 *	2.66*E* − 05	2.93
AA893518	*loc680367 *	0.00028	2.87
AI043805		3.83*E* − 06	2.77
AB039826	*loc259245 /// loc259246 /// mup5 *	8.02*E* − 07	2.68
U46118	*cyp3a9 *	2.99*E* − 05	2.52
AB039823	*loc259246 *	3.95*E* − 07	2.51
NM_012693	*cyp2a2 *	1*E* − 06	2.45
NM_032082	*hao2 *	6.58*E* − 05	2.36
NM_012883	*sult1e1 *	1.81*E* − 05	2.34
NM_134380	*ust5r *	7*E* − 05	2.20
AI072107	*akr1c1 *	9.61*E* − 05	2.16
AB039828	*mup5 *	0.000142	2.10
BM385735	*stac3 *	4.67*E* − 05	2.07
NM_019184	*cyp2c29 *	4.09*E* − 05	1.96
NM_053524	*nox4 *	0.000186	1.88
AF072439	*zfp37 *	9.94*E* − 05	1.75
AW523958		0.001031	1.73
AI232716	*inmt *	0.000633	1.71
AW521319		1.35*E* − 05	1.70
BM390462	*rgd1310209 *	1.06*E* − 05	1.68
BF417649		5.11*E* − 06	1.68
BI289963		1.2*E* − 05	1.67
AI454016	*lrtm2 *	0.002522	1.66
BE110108	*dusp1 *	0.002989	1.66
AA866264	*rgd1564865 *	7.97*E* − 05	1.63
AI548958	*hrasls *	0.000412	1.63
AB039828	*mup5 *	0.004619	1.63
AI071674		0.000412	1.62
AA892888	*mettl7b *	3.29*E* − 06	1.62
BF395080		0.000385	1.61
AI136882	*rgd1560784 *	0.000154	1.60
NM_053999	*ppp2r2a *	9.91*E* − 06	1.60
NM_058208	*socs2 *	0.000126	1.60
BF289150		5.23*E* − 05	1.60
NM_053977	*cdh17 *	0.027013	1.58
AW434139		9.35*E* − 08	1.58
NM_012608	*mme *	0.000567	1.57
BG375383	*rgd1308116* (*nim1*)	3.05*E* − 05	1.55
AI716500		0.001038	1.54
BF414998	*rgd1306105* (*tmem150c*)	9.72*E* − 05	1.54
AJ243338	*rt1-ce5 *	0.090847	1.53
BF558981	*pcdh17 *	0.004396	0.67
NM_031572	*cyp2c12 *	6.21*E* − 05	0.66
NM_130414	*abcg8 *	0.004836	0.66
NM_012753	*cyp17a1 *	2.67*E* − 05	0.66
NM_033352	*abcd2 *	0.000309	0.66
AA998516	*ccna2 *	0.000703	0.66
U07202	*asns *	0.00178	0.65
BI281851	*loc685203 *	0.002673	0.64
NM_012488	*a2m *	0.000111	0.64
NM_031050	*lum *	0.001029	0.64
NM_012521	*s100g *	0.016704	0.64
AA892854	*cxcl13 *	0.002336	0.64
AF062389	*acsm2 *	0.000189	0.63
BE112927	*cyfip2 *	0.00012	0.63
AI101139	*tox *	0.000121	0.63
NM_019157	*aqp7 *	0.000186	0.62
AI230228	*psat1 *	9.8*E* − 05	0.62
AW523490		0.001056	0.62
AI408151	*rgd1566215 *	2.5*E* − 05	0.60
AW252129	*nfe2 *	0.003234	0.60
BE116152	*elovl6 *	5.18*E* − 05	0.59
BM390001	*rgd1562284 *	0.000166	0.59
AF072816	*abcc3 *	0.000377	0.58
AI235528		4.13*E* − 05	0.56
BF284168		6.65*E* − 08	0.54
BF396857	*elovl6 *	0.000111	0.53
AI599365		0.00087	0.52
AA963228		0.000112	0.48
AI102401		0.000117	0.45
NM_053781	*akr1b7 *	0.000265	0.40

**Table 3:** Significant regulated pathways between adults and pups.

Pathway	Genes	*P*
Transport	*obp3*, *mup5*, *ust5r*, *mme*, *clca5*, *gpm6a*, *tmem163*; *abcg8*, *abcd2*, *aqp7*, *abcc3 *	0.001

Steroid	*obp3*, *hsd3b5*, *akr1c1*, *hsdl2*, *hsd17b6*, *hsd11b2*, *ar*; *cyp17a1 *	8.6*e* − 9

Metabolic process	*hsd3b5*, *dhrs7*, *hao2*, *mett17b*, *hsdl2; asns,acsm2,psat1 *	0.035

Redox	*hsd3b5, cyp2c13, cyp3a9, cyp2a2, hao2, akr1c1, cyp2c29, nox4, rgd1564865, hsdl2, me3; cyp2c12, cyp17a1, akr1b7 *	0.0001

Transcription regulation	*zfp37, zfp68, npas2; taf9b, ppargc1a, nfe2 *	0.0003

Cell adhesion	*cdh17, omd, jam3; pcdh17, ncam2, amigo1 *	0.005

Collagen	*;col4a1, col5a2, col5a1, col1a2, col1a1*	4.4*e* − 5

Immune response	*rt1-ce5, cxcl9, rt1-aw2, tnfsf13; cxcl13 *	0.099

Signaling	*ppp2r2a, socs2, olr59, rgs3, adora2b, cish, atp6ap2, pik3c3, ppp1r2, ghr, nrg1;*	0.00067

Organization	*cav2, pex11a, onecut1, meox2, cml3; col5a2,l ox, *	0.0051

Fatty acid biosynthesis	*scd, fasn;*	0.00014

Cyclin	*ccng2; ccnb2, ccna2 *	0.00018

Solute carrier (slc)	*41a2, 25a30, 9a3r1, 31a2; 13a3,1a3*, *4a1*,	0.0033

Proteolysis (peptidase)	*mup5*, *mme*, *trhde*, *spink3*, *prcp*; *rgd1562284 *	9.1*e* − 4

Ubiquitin	*hspb1*, *usp18*;	0.029

G protein	*gnai3 gnat3 adora2b*;	0.0094

Gene: higher; lower.

**Table:** (a) Cumulative comparison

Group	*Q* ^2^, permutation test						
	w10^#^	w13	w15	w17	w19	w21	w23
Control	0.759	0.807	0.669	0.730	0.779	0.790	0.885

^#^Comparison with w8.

**Table:** (b) Link and other comparisons

Group	10–13	13–15	15–17	17–19	19–21	21–23		
Control	0.992	0.497	**0.269**	**0.187**	**0.232**	0.807		

Group	10–15	10–17	10–19	10–21	10–23	13–17	13–19	

Control	**0.519**	0.793	0.497	0.544	0.906	0.613	0.896	

Group	13–21	13–23	15–19	15–21	15–23	17–21	17–23	19–23

Control	0.677	0.919	**<0 **	0.642	0.930	0.796	0.920	0.734

Bold: invalid model. Components: autofit.
